# Intravenous sildenafil for treatment of early pulmonary hypertension in preterm infants

**DOI:** 10.1038/s41598-023-35387-y

**Published:** 2023-05-24

**Authors:** Lukas Schroeder, Paulina Monno, Brigitte Strizek, Till Dresbach, Andreas Mueller, Florian Kipfmueller

**Affiliations:** 1 Department of Neonatology and Pediatric Intensive Care Medicine, University Children’s Hospital Bonn, Venusberg-Campus 1, 53127 Bonn, Germany; 2 Department of Obstetrics and Prenatal Medicine, University Hospital Bonn, Bonn, Germany

**Keywords:** Paediatric research, Cardiovascular diseases

## Abstract

Data is lacking on the effect of continuous intravenous sildenafil treatment in preterm infants with early pulmonary hypertension (PH), especially in very low birth weight (VLBW) infants. Preterm infants (< 37 weeks of gestational age) with intravenous sildenafil treatment and diagnosis of PH between 01/12 and 12/21 were retrospectively screened for analysis. The primary clinical endpoint was defined as response to sildenafil according to the improvement of the oxygenation index (OI), the saturation oxygenation pressure index (SOPI) and PaO_2_/FiO_2_-ratio. Early-PH was defined as diagnosis < 28 day of life (DOL). 58 infants were finally included, with 47% classified as very low birth weight (VLBW) infants. The primary endpoint was reached in 57%. The likelihood to die during in-hospital treatment was more than three times higher (72 vs 21%, p < 0.001) in infants without response to sildenafil. The echocardiographic severity of PH and right-ventricular dysfunction (RVD) decreased significantly from baseline to 24 h (p = 0.045, and p = 0.008, respectively). Sildenafil treatment leads to significant improvement of the oxygenation impairment in 57% of the preterm infants, with similar response rates in VLBW infants. Intravenous sildenafil treatment is associated with a significant decrease of the PH-severity and RVD.

## Introduction

Pulmonary hypertension (PH) in preterm and term newborns is a major contributor for short and long term morbidity and mortality in this cohort^1–6^. Mortality rates remain still high despite the development of new promising drugs for PH treatment over the last two decades. According to the updates and new classification system of PH on the 6th World Congress on Pulmonary Hypertension (Nice, 2018) PH in preterm and term infants is frequently related to: (1) Persistent PH of the newborn syndrome (PPHN, class I, group 1.7) and (2) PH due to lung disease; mostly in presence of bronchopulmonary dysplasia (BPD) associated PH (BPD-PH, class III)^7^. Early-PH is defined according to the occurrence in the first weeks of life (< 28 day of life-DOL). The incidence of early-PH in preterm infants was calculated with 24% in a recent Meta-analysis^8^. In contrast, in many preterm infants PH is diagnosed at later stages (diagnosis > 28 DOL) and the incidences of late-PH (including BPD-PH) are ranging between 5 and 25%^9–11^.

One candidate drug for the treatment of PH in preterm and term newborns is sildenafil. Sildenafil acts via the cyclic guanosine monophosphate (cGMP) pathway and as phosphodiesterase 5 (PDE 5) inhibitor, inhibiting PDE 5, which normally breaks down cGMP in the smooth muscle cells and therefore increases cytoplasmatic cGMP levels. This leads to a calcium-efflux and induces a smooth muscle cell relaxation, leading to pulmonary vasodilation^12^.

Sildenafil is available as intravenous (continuous and intermittent) and oral (sildenafil-citrate) form of administration. In recent years, several studies evaluated sildenafil as treatment for PH (BPD-PH, PPHN) in preterm and term infants in prospective trials^13–15^. The majority of the studies published to date and included in the Cochrane review of sildenafil treatment for PH (2017) were exclusively enrolling neonates with a GA > 34 weeks^16^. Despite the effort done by the studies conducted in the past, data on intravenous sildenafil treatment in preterm infants < 34 weeks and especially in VLBW infants with early PH are still lacking. Data from retrospective trials on sildenafil treatment in preterm infants are available, but predominantly focusing on oral drug administration^17,18^. More data is warranted analyzing data of intravenous sildenafil therapy in preterm infants in larger cohorts.

The aim of this study was to evaluate the data on continuous intravenous sildenafil treatment in preterm infants (< 37 weeks of GA) with diagnosis of early PH over the last decade (2012–2021), to generate more insight in the effect of intravenous sildenafil treatment for early PH.

## Material and methods

### Study population

Infants treated at the NICU of the University Children´s Hospital of Bonn, Germany, during the study period 01/2012–12/2021 were retrospectively screened for study participation. Inclusion criteria: echocardiographic verification of PH, continuous intravenous sildenafil treatment, < 37 weeks of GA. Exclusion criteria were as follows: ≥ 37 weeks of GA, congenital diaphragmatic hernia (a study analyzing continuous intravenous sildenafil treatment in this subgroup was previously published^19^), oral sildenafil treatment prior to intravenous treatment or exclusive oral treatment, primary palliative care, congenital heart defect requiring surgical repair.

### Ethical approval

The study was approved by the local ethics committee of the Medical Center of the University of Bonn (local running number 138/22). Informed consent was waived by decision of the ethics committee of the Medical Center of the University of Bonn due to the retrospective design of the study. The methods used for the clinical research were performed in accordance with relevant guidelines/regulation and in accordance with the Declaration of Helsinki.

### Monitoring data and oxygenation scores

The following hemodynamic parameters were retrospectively recorded from the patient´s chart at baseline, 3, 6, 9, 12, 24, 48 h, and at day 7 after start of sildenafil treatment: systolic, diastolic, and mean arterial blood pressure, heart rate, pre- and postductal SpO_2_, FiO_2_. Arterial blood gas measurements with pH, paO2, and paCO2 were recorded when available at baseline, 3, 6, 9, 12, 24, 48 h and day 7. The (a) Oxygenation Index (OI; $\frac{FiO2\, x\, Mean\, airway\, pressure \left(MAP\right)}{paO2}$) for infants with invasive mechanical ventilation (MV), (b) the Saturation Oxygenation Pressure Index (SOPI; $\frac{CPAP\, pressure\, or\, PEEP\, x \,FiO2}{SpO2}$)^20^ for infants without MV but with continuous positive airway pressure-CPAP or highflow-nasal cannula-HFNC support, and (c) PaO_2_/FiO_2_ (P/F)-ratio (for infants with both, MV or non-invasive support) were retrospectively calculated for the timepoints baseline, 12, 24 and 48 h to evaluate oxygenation impairment. The vasoactive-inotropic score (VIS) was calculated for the same timepoints according to the formula described elsewhere for estimation of cardiovascular drug support^21^.

### Diagnostic and treatment of pulmonary hypertension

PH was defined as evidence of echocardiographic signs of PH and classified as early PH when appearance was in the first 28 DOL. These definition were adopted from the definition of PH in preterm infants described elsewhere^10,11,22^. Echocardiographic PH assessment is described below in detail.

PH drug treatment was conducted by the attending physicians according to in-house standards of clinical practice for vasoactive and PH treatment. Inhaled nitric oxide (iNO) was used as primary drug therapy when PH was treated. Infants were evaluated for sildenafil therapy when echocardiographic assessment demonstrated moderate or severe PH, FiO_2_ was > 0.8 despite iNO treatment, and difference in pre- and postductal SpO_2_ was > 8% (in case of a patent ductus arteriosus-PDA). Sildenafil was administered routinely as continuous intravenous infusion and a dose regime of 1.6 mg/kg/day, as described previously and according to the in-house clinical standards^19,23,24^. A bolus infusion of 0.4 mg/kg over a period of 3 h was administered followed by a continuous infusion according to the decision of the attending physician. For the period of sildenafil administration all infants were treated simultaneously with iNO.

Infants with need for inotropic support were treated with dobutamine and with milrinone. Furthermore, levosimendan was administered in infants with cardiac dysfunction despite high-dose inotropic treatment. In cases of oxygenation failure unresponsive to conventional treatment, extracorporeal membrane oxygenation (ECMO) was implemented according to international guidelines and criteria^25,26^.

### Echocardiographic assessment

For echocardiographic measurements a Philips CX50 Compact Extreme Ultrasound system with a S12-4 sector array transducer (Philips Healthcare, Best, the Netherlands) was used. All infants with diagnosis of early or late PH were evaluated by an echocardiographic assessment daily or (when stabilized) every 48–72 h, according to the in-house standards of clinical practice. All echocardiographic data at baseline (prior to start of sildenafil administration), at 24 h, and 48 h were retrospectively screened for analysis independently by two experienced neonatal echocardiographers. Both echocardiographers have over 10 years’ experience in neonatal echocardiography as senior physicians, with profound knowledge of the echocardiographic diagnosis of PH and cardiac dysfunction in preterm and term infants in a tertiary referral medical center. PH was graded as mild, moderate, or severe, using the following echocardiographic parameters: (a) flow pattern of the ductus arteriosus (DA), (b) intraventricular septum position (IVS), and (c) tricuspid valve regurgitation (TVR). Tricuspid valve regurgitation was graded as I°, II° or III°. Mild PH was diagnosed as follows: DA shunt-flow was left-to-right, IVS was flattened, and TVR was I°–II°. Moderate PH was diagnosed as follows: DA shunt-flow was alternating (left-to-right/ right-to-left), IVS was flattened, and TRV was II–III°. Severe PH was diagnosed as follows: DA shunt-flow was right-to-left, IVS was D-shaped (towards left ventricular cavity), and TRV was III°. Additionally, the end-diastolic right-ventricular to left-ventricular (RV/LV) ratio was calculated in a standard four chamber directly distal to the tricuspid and mitral annulus as a horizontal line from endocardium of the RV and LV free wall to endocardium of the interventricular septum. Presence of ventricular dysfunction was defined as (a) right-ventricular dysfunction (RVD) and (b) left-ventricular dysfunction (LVD). For the assessment of ventricular dysfunction a combined approach of quantitative and qualitative measurements was used, based on international guidelines^27,28^.

### Statistical analysis and outcome measures

Infants were retrospectively classified as sildenafil treatment Responder and non-Responder. A response to sildenafil treatment (primary outcome parameter) was defined as: decrease of the OI ≥ 20% (infants with MV) or the SOPI (infants without MV, but with non-invasive support) or an increase of the P/F ratio ≥ 20% (both, infants with and without MV) within the first 12–24 h after sildenafil. The following parameters were defined as secondary outcome measures: decrease of PH severity in the echocardiographic assessment during sildenafil therapy at timepoints 24 and 48 h, duration of MV, days of oxygen supplementation, oxygen supplementation at discharge, diagnosis of bronchopulmonary dysplasia (BPD) at 36 weeks of GA, survival to discharge.

For this descriptive analysis, continuous variables were described using median and interquartile range (IQR). Categorical variables were summarized as absolute number (n) and percentage. For comparison of continuous and non-normally distributed variables, a Wilcoxon-test or Mann–Whitney U test was performed to compare continuous variables between timepoints and subgroups (Responder vs non-Responder), as appropriate. For categorical variables the Pearson’s Chi^2^ test and Fisher’s exact test was applied, as appropriate.A Kaplan–Meier plot was used to analyze the survival chance after start of sildenafil treatment. A p-value of < 0.05 was considered significant.

## Results

In total, 75 infants with documented diagnosis of PH and intravenous sildenafil treatment in the electronic patient´s chart system were screened for inclusion and 58 infants were finally enrolled for the analysis. 17 infants were excluded due to the following criteria: in 6 infants the patients’ charts, and documentation was incomplete, in 9 infants’ sildenafil was administered oral prior to the continuous intravenous infusion, and 2 infants had a diagnosis of late-onset PH (> 28 DOL).

Epidemiological data of the cohort are displayed in Table 1. The primary endpoint (response to sildenafil therapy) was reached in 57% (n = 33), with a response rate of 54% in VLBW infants and 59% in infants with a birth weight > 1500 g. Sildenafil was administered in median at the 2 (1/3) DOL. Almost the half of the cohort (n = 26, 45%) were classified as VLBW infants and 14 (24%) was born with < 28 weeks of GA (extremely low gestational age newborns-ELGAN).

**Table 1:** Demographic and treatment data.

Variables	Overall cohort n = 58	Responder n = 33	Non-Responder n = 25	p-level
Gestational age, w	31.8 (28.5/34.3)	32.3 (28/34.9)	31.4 (29.2/34.1)	0.832
Female sex, n (%)	30 (51)	14 (42)	16 (64)	0.120
Birth weight, kg	1.8 (0.9/2.5)	1.8 (0.9/2.5)	1.7 (0.8/2.4)	0.423
APGAR 5 min	7 (6/8)	7.5 (6/8)	7 (5.5/8)	0.187
APGAR 10 min	8 (7/9)	8 (7/9)	8 (7/9)	0.346
CRIP-Score	11.5 (9.3/14)	11 (8.8/15.3)	13.5 (10/14)	0.585
Lowest FiO2 in the first 24 h	0.28 (0.21/0.66)	0.35 (0.21/0.75)	0.26 (0.21/0.4)	0.354
Primary diagnosis, n (%)				
(a) Twin-to-Twin-transfusion syndrome, n (%)	5 (9)	2 (6)	3 (12)	0.643
(b) Fetal hydrops, n (%)	11 (19)	6 (18)	5 (20)	0.99
(c) Fetal growth retardation, n (%)	13 (22)	7 (21)	6 (24)	0.99
(d) Lung hypoplasia	25 (43)	8 (24)	17 (68)	**0.001**
(e) Lower urinary tract obstruction (LUTO) or congenital renal disorder	9 (16)	2 (6)	7 (28)	**0.031**
(f) Genetic disorder	8 (14)	3 (9)	5 (20)	0.272
Comorbidities, n (%)				
BPD at 36 weeks of gestational age	15 (26)	12 (36)	3 (12)	**0.030**
Intraventricular hemorrhage	13 (22)	8 (24)	5 (20)	0.700
Necrotizing enterocolitis	18 (31)	10 (30)	8 (32)	0.99
Retinopathy of prematurity, grade ≥ 2	10 (17)	6 (18)	4 (16)	0.99
Sepsis	15 (26)	7 (21)	8 (32)	0.381
Mechanical ventilation, d	2.3 (0.1/22.5)	5.8 (0.7/28)	1.1 (0.1/16.5)	0.142
Oxygen supplementation, d	8 (3/36)	9 (4/52)	5 (2/23)	0.285
In-hospital mortality, n (%)	25 (43)	7 (21)	18 (72)	** < 0.001**
In-hospital mortality or BPD, n (%)	38 (66)	18 (55)	20 (80)	**0.05**
ECMO support, n (%)	4 (7)	3 (9)	1 (4)	0.627

Data are demonstrated as absolute number with percentage or as median values with IQR. Infants with a decrease of ≥ 20% of the Oxygenation Index (OI), the Saturation Oxygenation Pressure Index (SOPI) or an increase ≥ 20% of the PaO2/FiO2 ratio (P/F ratio) were defined as responder to sildenafil. Parameters with a p-level < 0.05 are highlighted in bold.

BPD, bronchopulmonary dysplasia at 36 weeks of gestational age; CRIP, clinical risk index for babies; ECMO, extracorporeal membrane oxygenation).

**Figure 1:**
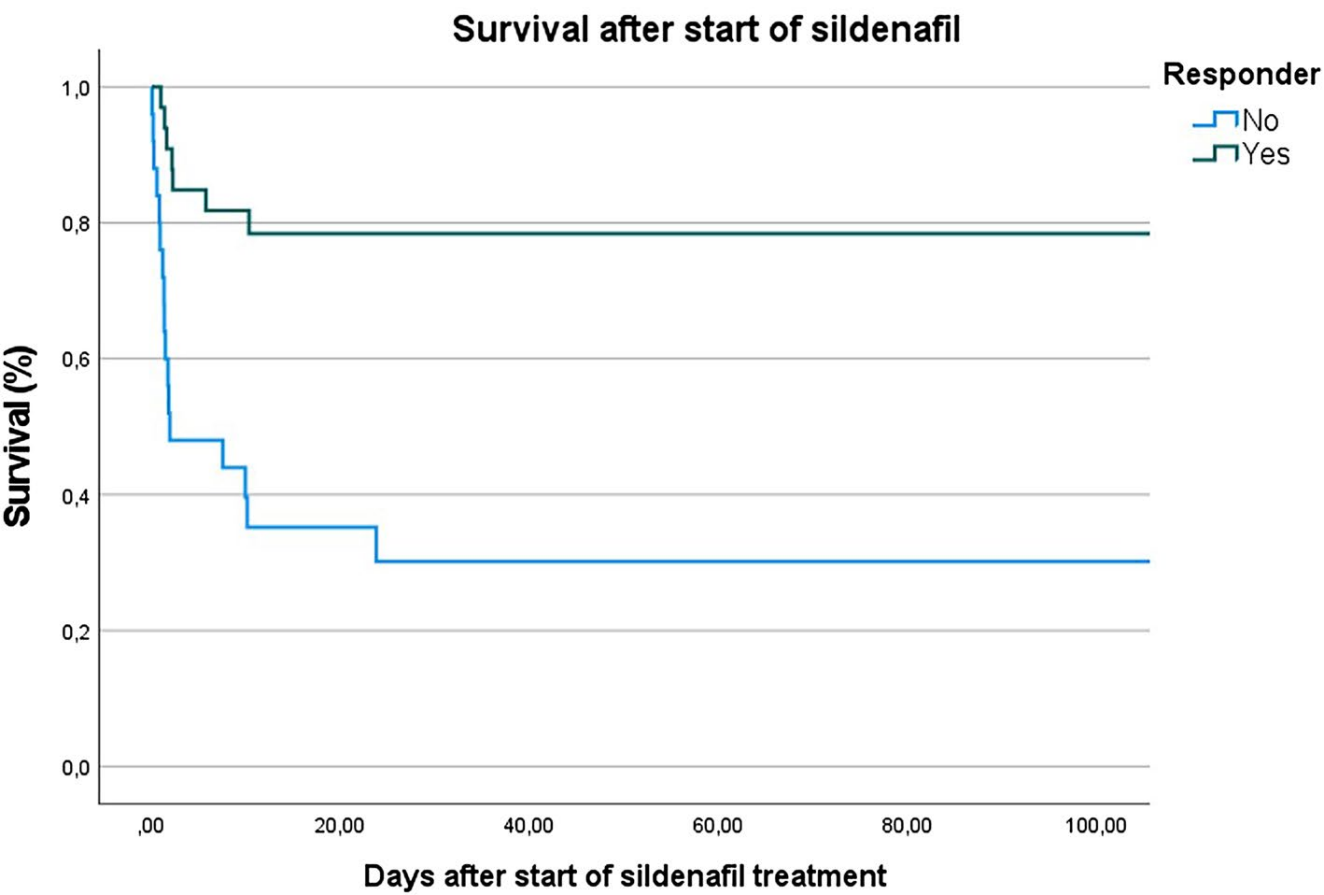
The Kaplan–Meier plot displays the survival rate after start of sildenafil treatment. The non-Responder infants are illustrated with the blue line, the Responder infants are illustrated with the green line.

Significantly more infants in the non-Responder group suffered from a lung-hypoplasia (p = 0.001) and a lower urinary tract obstruction (LUTO) or renal congenital disorder (p = 0.031) at birth. Lung hypoplasia was defined according to the prenatal diagnosis by the obstetricians or clinical/radiological signs after birth (severe respiratory insufficiency with X-ray findings as small lung fields, diaphragmatic domes, and a bell-shaped)^29^. The rates of comorbidities after birth were equally distributed between the subgroups. The mortality rate was 43% in the overall cohort, with a significantly higher mortality rate in the non-Responder group (21 vs 72%, p < 0.001, see Kaplan–Meier-plot in Fig. 1). When looking for the combined endpoint death and/or BPD there between Responder and non-Responder infants there is still a trend to a higher rate of the combined endpoint in non-Responder infants (see Table 1, p = 0.05). Baseline characteristics in the first 24 h (APGAR 5 and 10 min, CRIP score and lowest FiO2) were similar between the subgroups.

### Oxygenation scores and treatment data

**Figure 2:**
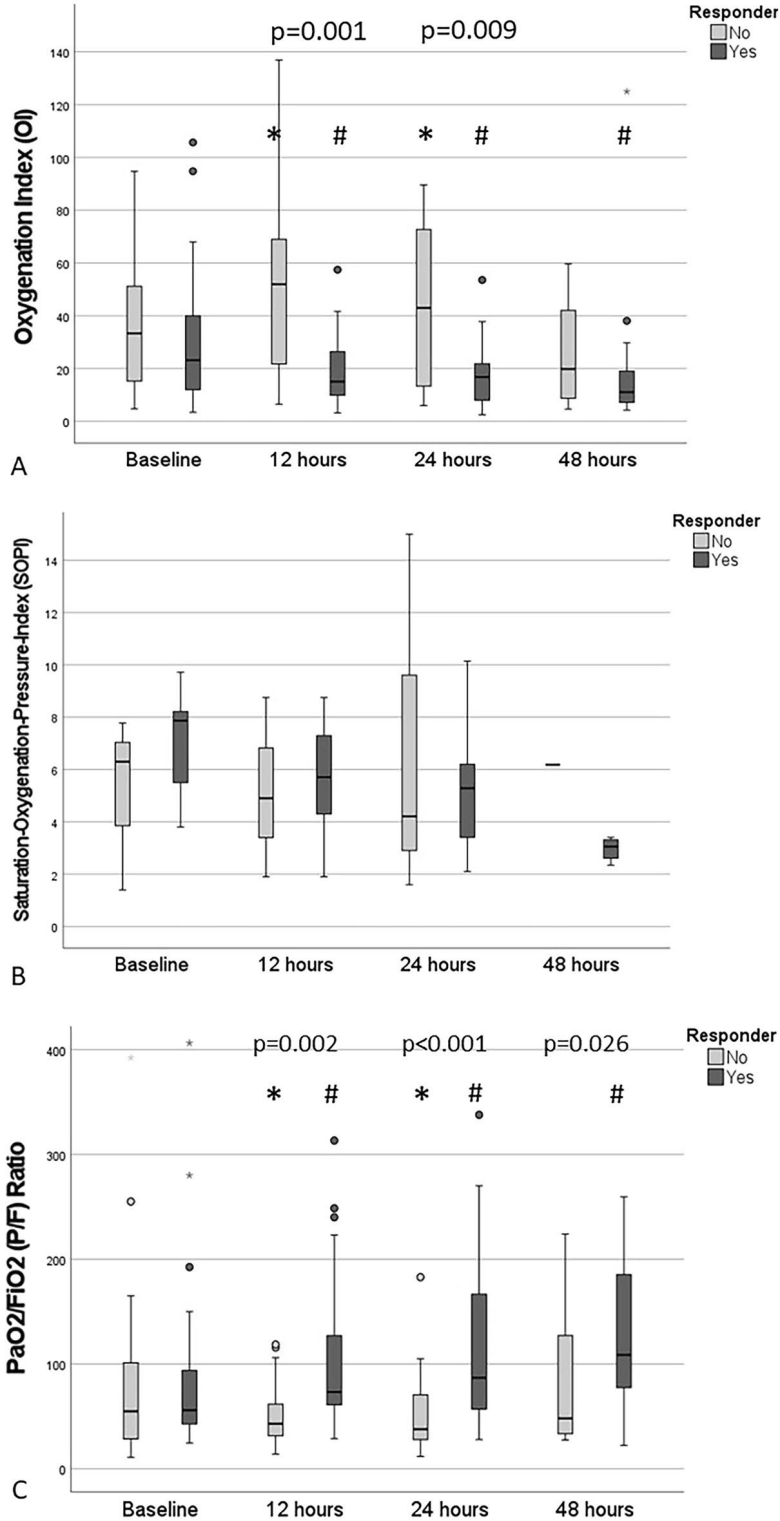
(**A**) The mean values for the Oxygenation Index (OI) were calculated for the subgroups (Responder vs. non-Responder). (**B**) The mean values for the Saturation Oxygenation Pressure Index (SOPI) were calculated for the subgroups (Responder vs. non-Responder). (**C**) The mean values for the PaO_2_/FiO_2_ (P/F) ratio were calculated for the subgroups (Responder vs. non-Responder). All values were calculated at baseline (prior to sildenafil treatment start), at 12 h, 24 h, and 48 h after sildenafil treatment start. p-values pointed out in the figures are related to significant differences between subgroups. The asterisk and the rhombus illustrate p-levels < 0.05, when comparing the value with the respective baseline value in the subgroup.

The course of oxygenation according to the oxygenation scores (OI, SOPI and P/F-ratio) and allocation to the Responder and non-Responder group with related statistical significances is displayed in Fig. 2A–C.

**Figure 3:**
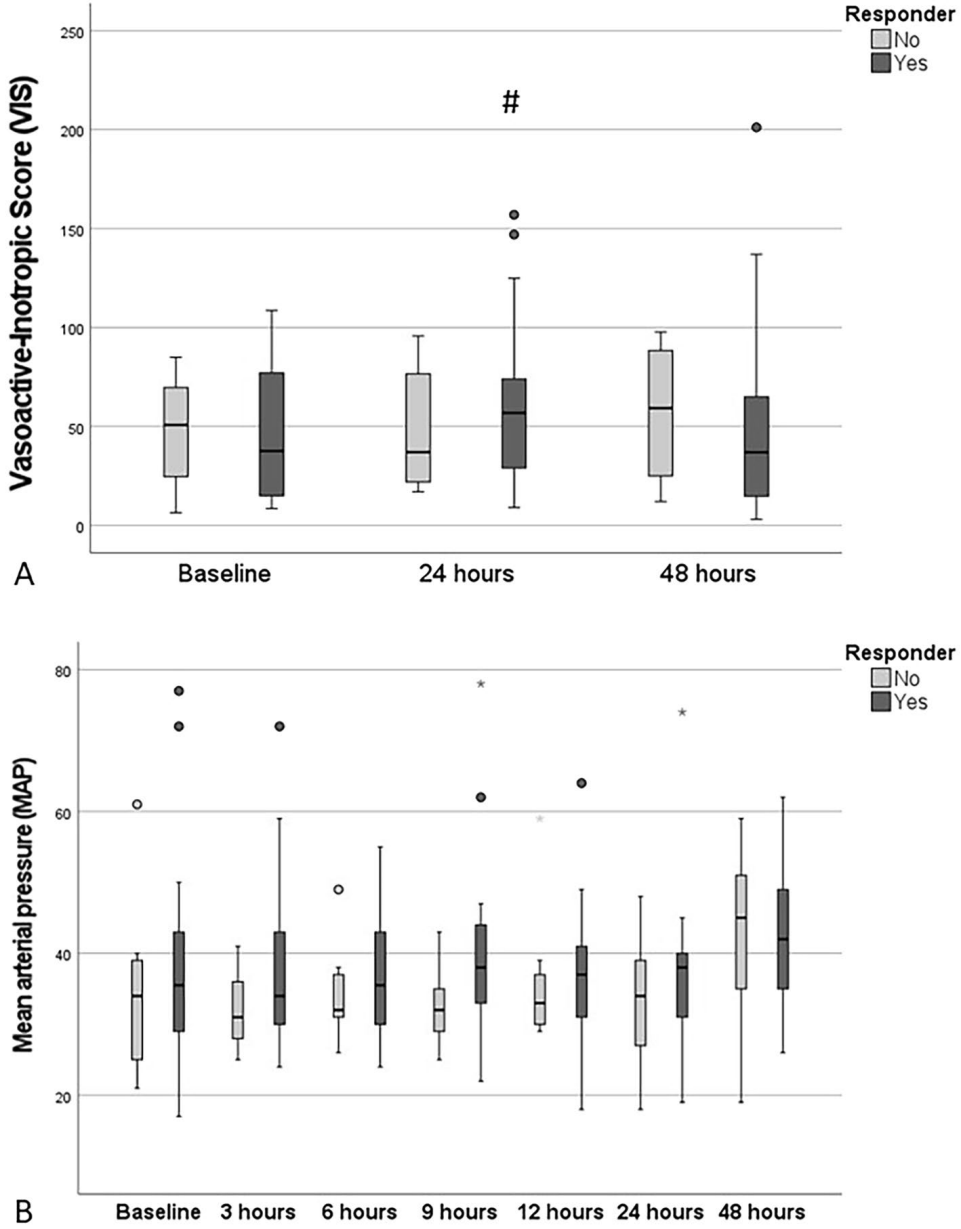
(**A**) The Vasoactive-Inotropic Score (VIS) was calculated for the respective subgroups (Responder vs. non-Responder) at baseline, 24 h, and 48 h. (**B**) The mean arterial pressure (MAP) was documented from the patient´s chart and electronic documentation system every 3 h ongoing from baseline to 48 h. The rhombus illustrates p-levels < 0.05, when comparing the value with the respective baseline value in the subgroup.

The mean VIS scores after sildenafil treatment start are illustrated in Fig. 3A. In the Responder group, the mean VIS score increased significantly from baseline to 24 h (p = 0.004), without significant changes in the non-Responder group. The mean arterial pressure (MAP, see Fig. 3B) tended to increase during the sildenafil treatment. After commencing sildenafil the median arterial lactate levels decreased significantly from baseline (2.95 mmol/l) to timepoint 48 h (2.19 mmol/l, p = 0.037). No difference was found between Responder and non-Responder.

Comparison of outcome and treatment data between subgroups are illustrated in Table 2**.** No difference was found regarding the use of concomitant drugs for PH (iNO, bosentan or levosimendan) between Responder and non-Responder.

**Table 2:** Treatment data.

Variables	Overall cohort n = 58	Responder n = 33	Non-Responder n = 25	p-level
Sildenafil treatment data				
Start of i.v. sildenafil, DOL	2 (1/3)	2.5 (1/3)	2 (1/2.5)	0.344
Stop of i.v. sildenafil, DOL	7 (3/13)	8 (6/15)	5 (2/12.5)	0.057
Duration of i.v. sildenafil, h	120 (26/216)	141 (53/233)	41 (11/216)	0.067
Sildenafil Bolus at start of infusion (0.4 mg/kg, 3 h)	29 (50)	16 (48)	13 (52)	0.793
Dose duplication of sildenafil to 3.2 mg/kg/d after cessation of bolus infusion, n (%)	4 (8)	3 (10)	1 (5)	0.99
Oral sildenafil treatment during NICU stay, n (%)	21 (36)	16 (49)	5 (20)	**0.031**
Discharge with oral sildenafil treatment, n (%)	20 (35)	12 (36)	8 (32)	0.786
Concomitant treatment data				
iNO treatment at start of sildenafil therapy, n (%)	55 (95)	32 (97)	23 (92)	0.572
Duration of iNO treatment, h	115 (29/291)	123 (54/355)	37 (20/241)	0.083
Bosentan treatment after start of sildenafil, n (%)	8 (14)	6 (18)	2 (8)	0.445
Dobutamine dose at start of sildenafil treatment, µg/kg/min	10 (5/12)	9 (5/11)	10 (5/14)	0.456
Dobutamine dose at 24 h of sildenafil treatment, µg/kg/min	10 (6/10)	10 (5/10)	10 (8/13)	0.418
Milrinone dose at start of sildenafil treatment, µg/kg/min	0.69 (0.46/0.70)	0.7 (0.5/0.7)	0.66 (0.3/0.7)	0.360
Milrinone dose at 24 h of sildenafil treatment, µg/kg/min	0.70 (0.67/0.70)	0.7 (0.67/0.7)	0.7 (0.67/0.7)	0.928
Norepinephrine dose at start of sildenafil treatment, µg/kg/min	0.45 (0.2/0.5)	0.5 (0.18/0.5)	0.4 (0.2/0.5)	0.901
Norepinephrine dose at 24 h of sildenafil treatment, µg/kg/min	0.5 (0.25 /0.65)	0.5 (0.34/0.72)	0.5 (0.17/0.6)	0.588
Vasopressin dose at start of sildenafil treatment, mU/kg/min	0.7 (0.4/1.6)	0.6 (0.4/1.4)	0.9 (0.4/1.9)	0.489
Vasopressin dose at 24 h of sildenafil treatment, mU/kg/min	1.2 (0.6/2.3)	1.1 (0.6/2.1)	1.4 (0.6/4.0)	0.431
Levosimendan (0.2 µg/kg/min) treatment at start of sildenafil therapy, n (%)	6 (10)	3 (9)	3 (12)	0.99
Mechanical ventilation at start of sildenafil treatment, n (%)	48 (83)	26 (79)	22 (88)	0.490
Discharge with oxygen supplementation, n (%)	32 (59)	14 (45)	18 (78)	**0.024**

Data are presented as median with IQR or absolute number with %. A p-value < 0.05 was considered as statistically significant and are highlighted in bold letters.

d, days; CPAP, continuous positive airway pressure; DOL, day of life; h, hours; iNO, inhaled nitric oxide; i.v., intravenous; n, number; NICU, neonatal intensive care unit).

### Echocardiographic assessment

**Figure 4:**
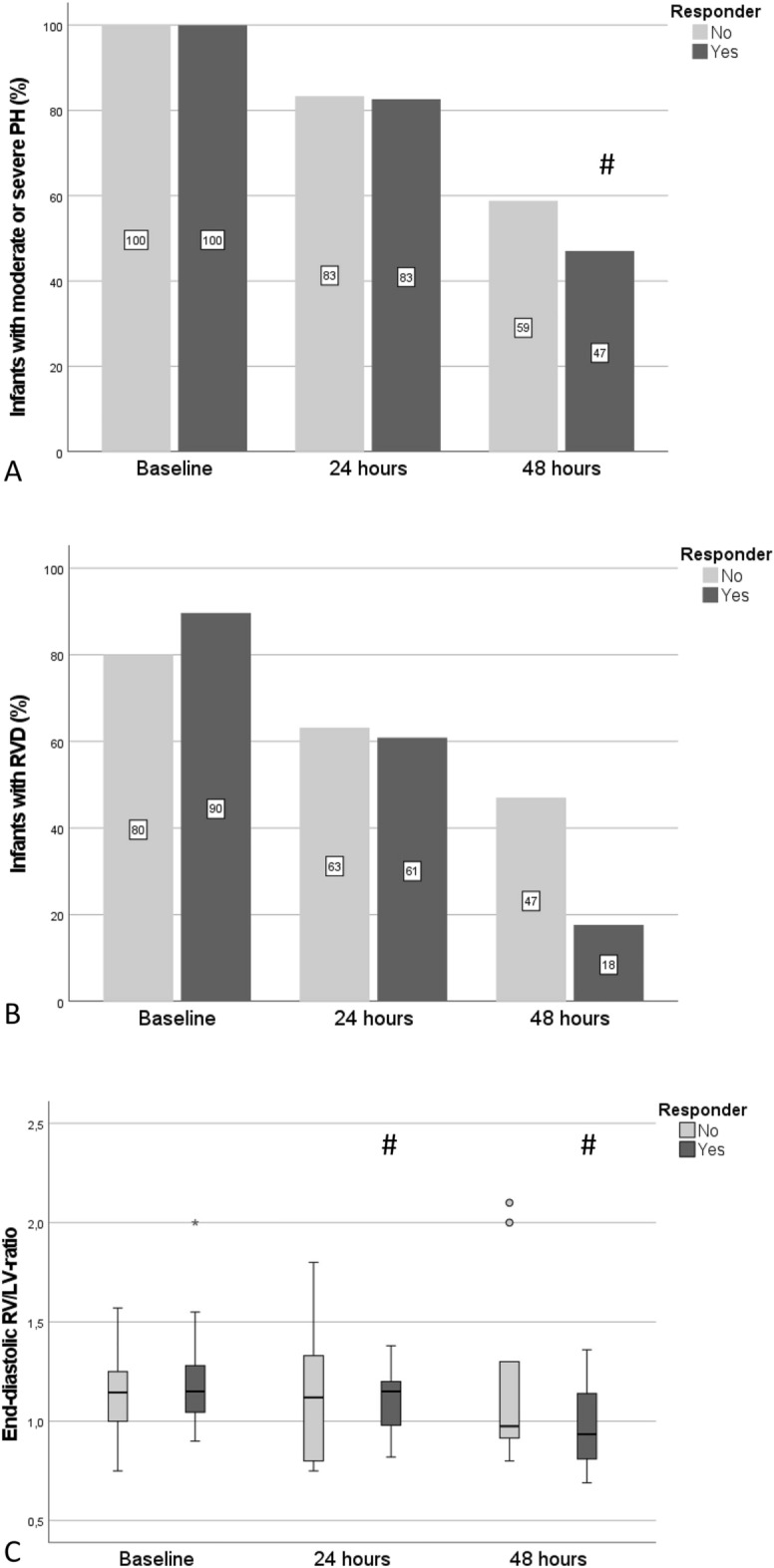
(**A**) The severity of PH (moderate or severe) during the sildenafil treatment was assessed according to the echocardiographic data for the timepoints baseline (prior to treatment), 24 and 48 h (after treatment start). (**B**) The incidence of the RVD (present/ not present) during the sildenafil treatment was assessed according to the echocardiographic data for the timepoints baseline (prior to treatment), 24 and 48 h (after treatment start). (**C**) The RV/LV ratio during the sildenafil treatment was calculated according to the echocardiographic data for the timepoints baseline (prior to start), 24 and 48 h (after treatment start). The rhombus illustrates p-levels < 0.05, when comparing the value with the respective baseline value in the subgroup.

In 42 infants (71%) valid echo data were available at baseline and 24 h. Echo data at all three timepoints (+ 48 h) were available in 35 infants (59%). At baseline, 93% of the infants presented a persistent ductus arteriosus (PDA), with 33% presenting a right-to-left shunt over the PDA. At 24 h 95% of the infants presented a PDA (19% with right-to-left shunt). The course of PH, RVD and end-diastolic RV/LV-ratio is illustrated in Fig. 4A–C. In the overall cohort, the severity of PH and RVD decreased significantly from baseline to 24 h (p = 0.045, and p = 0.008, respectively). The RV/LV-ratio decreased significantly in the Responder group from baseline to 24 h (p = 0.028), without statistically significant difference in the overall cohort. Regarding the presence of LVD at baseline, 24 h and 48 h no differences were found between the Responder and non-Responder group.

## Discussion

The present study summarizes a retrospective data set of 58 preterm infants with continuous intravenous sildenafil treatment and represents the biggest cohort to date of preterm infants with intravenous sildenafil treatment of early-PH. According to our results, we could demonstrate that PH severity, the incidence of RVD and the end-diastolic RV/LV ratio decreased significantly during the first 24 h during sildenafil treatment. Sildenafil treatment is associated with a rapid improvement in oxygenation impairment in the mayor part of the infants, illustrated and evaluated with established oxygenation indices (OI, SOPI, and P/F ratio), both shown in VLBW and non-VLBW preterm infants. The mortality rate of infants responding to sildenafil was significantly lower compared to non-Responder. Nevertheless, about 40% of the preterm infants did not demonstrate a response to sildenafil treatment and were classified as non-Responder.

In recent prospective trials intravenous sildenafil treatment was evaluated in neonates with a gestational age > 34 weeks, but data on intravenous sildenafil in ELGAN and VLBW preterm infants < 28 GA are still lacking^13,14^. Several studies analyzed oral sildenafil treatment and only two recent studies were identified analyzing intravenous sildenafil treatment including VLBW infants with PH^15,17,30,31^. In most of these infants PH was diagnosed secondary to the timepoint of BPD diagnosis (36 weeks of GA). These studies overall report beneficial effects of sildenafil on echocardiographic grading of BPD-PH and oxygenation indices. A prospective multicenter, randomized, placebo-controlled trial on preterm infants < 29 weeks of GA at risk for PH is ongoing (SILDI-SAFE trial)^32^. Our study adds new insights on preterm and VLBW-infants with sildenafil treatment for primary early-PH in the first days of life, and with almost a third of the population classified as ELGANs and nearly half of the cohort classified as VLBW infants.

The response to sildenafil according to the improvement in oxygenation indices in our cohort was documented in 57% of the infants, without influence of GA or birth weight on the response to sildenafil. The updated Cochrane review from 2017 on sildenafil treatment for PH in neonates highlighted, that sildenafil is effective in reducing mortality compared to placebo, with a number needed to treat of 3 patients to have a beneficial outcome^16^. Additionally, oxygenation indices improved in the first 24 h after administration of sildenafil. Nevertheless, these data are summarizing the effect of an oral sildenafil treatment. The most recent trial on intravenous continuous sildenafil was published in 2021 by Pierce et al. The study group conducted a multinational, double-blind, placebo-controlled trial, and randomized infants (> 34 weeks of GA) with PPHN or at risk for PPHN to receive either intravenous sildenafil (0.1 mg/kg bolus infusion over 30 min, followed by 0.03 mg/kg/h continuous infusion) or placebo (0.9% saline or 10% dextrose)^13^. All infants were on iNO treatment, which were the same conditions as in our study population. The authors concluded that intravenous sildenafil was not superior to placebo regarding the endpoints: treatment failure rate (need for additional drug for PPHN therapy, need for ECMO, or death prior to discharge, and time on iNO after starting sildenafil in infants without treatment failure). This phase III study raises the question, whether continuous intravenous sildenafil is effective to treat PPHN in preterm and term infants. However, the findings of the study are contrarious to other studies revealing a significant effect of intravenous sildenafil in this population^14,23^. The dose regime of sildenafil used in the phase III trial was half of the dose which was described by Steinhorn et al.^23^, using a bolus infusion of 0.4 mg/kg over 3 h, followed by a maintenance dose of 1.6 mg/kg/day. The missing effect between placebo and sildenafil could potentially be biased by lower plasmatic sildenafil levels due to lower concentrations of the bolus and continuous infusion, and in total cumulative half of the dose used in the open-label, dose-escalation trial by Steinhorn et al.

In our study, infants were treated with the dose regime provided by Steinhorn et al., but a bolus infusion was only administered in 50% of the infants, due to the decision of the attending physician. The severity of oxygenation impairment and PPHN in our cohort is comparable with the before mentioned studies, as the median OI at baseline prior to sildenafil start in our cohort was 26.2 (12.0/47.3) and both studies predominantly included infants with an OI > 15 and < 60. In 4 infants of our cohort the continuous sildenafil dose was doubled to 3.2 mg/kg/day after cessation of the bolus infusion as rescue procedure in case of severe PPHN or oxygenation failure, without difference in response to sildenafil between subgroups (see Table 2).

The preterm infants screened for this retrospective analysis were predominantly those with a delayed cardiopulmonary adaptation and impaired transition of the fetal circulation. As reported recently, preterm infants with early-PH and delayed cardiopulmonary adaptation (PH diagnosis > 48–72 h) are at risk for adverse outcome and BPD, as highlighted recently^33^. This is in line with our findings, with an overall rate of BPD of 26% in the cohort and a rate of 36% of those responding to sildenafil and surviving to discharge.

Non-Responder in our cohort suffered significantly more often from lung-hypoplasia, and LUTO or congenital-renal-disorder. Infants with LUTO or congenital-renal-disorder mainly suffer from lung-hypoplasia due to oligo- or anhydramnion. Preterm infants suffering from lung-hypoplasia seem to be prone to respond less to sildenafil, which potentially can be explained by a higher degree of structural lung abnormality (vasculopathy: excessive muscularization, intima thickening, rarefaction of pulmonary arteries and vessels) and higher grade of oxygen impairment at baseline. As sildenafil is a second-line PH-therapy, the use of sildenafil in these infants needs to be evaluated carefully. The term “lung hypoplasia” is not standardized and clear definitions are missing in the current era. Therefore, this diagnosis is to a certain degree subjective.

Our results illustrate, that intravenous sildenafil treatment is associated with a significant and rapid reduction of the PH severity. This is in line with previous studies on infants with PPHN and neonates suffering from PH due to congenital lung disease or BPD-PH^15,17,19,30,31^. According to our results, the RVD improves significantly during the continuous sildenafil infusion. Several studies analyzed the relationship between sildenafil treatment and right ventricular diastolic and systolic function, as shown by magnet resonance tomography scans, in right heart catheterization and in animal models for right ventricular dysfunction^34–38^. In summary, sildenafil has afterload-reducing effects with improvement of RV unloading, leads to an improvement of diastolic RVD, improves RV myocardial remodeling due to upregulation of gene-markers for hypertrophy and inflammation, and there are conflicting results regarding the improvement of the RV systolic function. During the sildenafil treatment the RV/LV ratio decreases significantly, potentially illustrating the effect of RV unloading and higher preload of the left-ventricle in preterm infants. The incidence of LVD initially decreased from baseline to 24 h in non-Responder, followed by a significant increase from 24 to 48 h after start of sildenafil treatment in non-Responder. Sildenafil possibly can lead to a deterioration of a preexisting LV dysfunction, due to a aggravation of the LV dysfunction caused by a higher LV preload and filling pressures, which is in line with previous findings of sildenafil treatment in infants with a congenital diaphragmatic hernia^19^. On the other hand, results from adult RCT trials revealed beneficial effects of sildenafil on LV function^39^.

### Limitations

Our data provide important information in these patient population and we hypothesize that continuous sildenafil treatment in preterm infants with early-PH seems to be well tolerated. However, this need to be interpreted carefully, as the frequent use of concomitant vasoactive treatment could have mitigated an arterial hypotension as drug related adverse event during the sildenafil treatment. During the treatment period in 9 infants a severe arterial hypotension (5 mmHg < GA for > 60 min) was identified in the documentation system, but these episodes were not related to the sildenafil treatment, as the episodes occurred unrelated to the start of the bolus or continuous sildenafil infusion. The present study was conducted retrospectively and not designed to evaluate the safety profile of sildenafil. This needs to be considered when planning future prospective randomized-controlled trials.

A comparator cohort is missing and as mentioned before, prospective randomized trials are warranted. Therefore, it is important to get knowledge from retrospective studies as provided by our results. Furthermore, the interpretation of the echocardiographic data is at risk for bias because echocardiographic assessment is based to some extent on a qualitative grading. Finally, sildenafil treatment was started as second-line therapy in critically ill infants with a high-degree of preexisting comorbidities. This can have biased the incidence of the response rate of infants to sildenafil treatment.

## Conclusion

Continuous intravenous sildenafil treatment is associated with a significant improvement of the oxygenation impairment in preterm infants, including ELGAN and VLBW infants. Additionally, continuous intravenous sildenafil treatment is associated with an improvement of PH, RVD and RV/LV ratio in the echocardiographic assessment in this population. Nevertheless, a substantial proportion (43%) of preterm infants do not respond to sildenafil, especially infants with prenatal diagnosed lung-hypoplasia. These infants are at high risk for in-hospital mortality.

## Data Availability

The data that support the findings of this study are available on request from the corresponding author. The data are not publicly available due to privacy or ethical restrictions.
